# Flow state and autonomic response patterns during sensory rejection tasks using the Uchida-Kraepelin Test

**DOI:** 10.1371/journal.pone.0335711

**Published:** 2025-10-30

**Authors:** Hiroyuki Kuraoka, Mitsuo Hinoue, Chikamune Wada, Shinji Miyake

**Affiliations:** 1 Department of Information Systems Engineering, Faculty of Science and Technology, Chitose Institute of Science and Technology, 758-65 Bibi, Chitose, Hokkaido, Japan; 2 Department of Occupational Hygiene, School of Health Sciences, University of Occupational and Environmental Health, 1-1, Iseigaoka, Yahatanishi, Kitakyushu, Fukuoka, Japan; 3 Graduate School of Life Science and Systems Engineering, Kyushu Institute of Technology, 2-4 Hibikino, Wakamatsu, Kitakyushu, Fukuoka, Japan; 4 Graduate School of Science and Technology, Chitose Institute of Science and Technology, 758-65 Bibi, Chitose, Hokkaido, Japan; Anhui Polytechnic University, CHINA

## Abstract

This study investigated the relationship between flow state and autonomic nervous system activity indices in 18 healthy male participants using a mental arithmetic task (Uchida-Kraepelin [U-K] test)—known as a sensory rejection task. The experiment consisted of two sets, each comprising a 5-minute rest period, followed by a 15-minute task period with varying task conditions of self-paced, to be performed at own pace, and competitive, as per the instruction “Always do as many calculations as possible, aiming to exceed the preceding performance.” In the subjective assessment, the flow, time perception, subjective mental workload, and feelings of fatigue were evaluated. Autonomic nervous system activity indices were continuously monitored. The results indicate that the U-K test, which is a low-difficulty, monotonous task, failed to induce a flow state. Physiological responses to mental tasks showed an increased heart rate, suggesting a Pattern 1 response. The participants who entered a flow state, based on their flow and time perception scores, had a decreased heart rate immediately after the task, supporting the association between the flow state and Pattern 2 responses. Considering the relationship between flow and Pattern 2 responses, it is recommended to assess flow using multiple physiological markers, including blood pressure.

## Introduction

Recent years have seen a global shift toward enhancing workers’ well-being, with a growing emphasis on positive psychological constructs such as work engagement and flow. Work engagement is characterized by a sustained and stable psychological state encompassing three key dimensions: 1) *vigor*, which refers to the maintenance of high levels of energy and mental resilience during work; 2) *dedication*, which involves a sense of pride, enthusiasm, and perceived significance of one’s work; and 3) *absorption*, defined as being fully concentrated and deeply engrossed in one’s tasks [1,2]. A prominent theory explaining “absorption” is Csikszentmihalyi’s flow theory. Flow is a comprehensive psychological state characterized by complete immersion in an activity in which individuals experience a loss of temporal perception and diminished self-awareness [3]. According to Csikszentmihalyi’s flow model [4], a flow state is achieved when individuals encounter situations in which the level of challenge matches their skill level. Furthermore, achieving a flow state enhances enjoyment and satisfaction with the task, which is attributed to deep absorption in the ongoing activity [5]. Therefore, flow is an optimal psychological state that promotes self-development by facilitating deep enjoyment and fulfillment. In this state, individuals become so absorbed in the activity that they lose track of time while simultaneously enhancing their skills as they work toward goal attainment. In other words, when individuals are deeply focused and significantly progress in a task, such as office work, they can be considered to be in a state of flow. A survey on flow among 113 workers demonstrated that this state is associated with job performance, with the relationship being particularly pronounced among employees with high levels of conscientiousness [6]. In addition, research examining programming tasks has reported that individuals with higher levels of intellectual curiosity tend to have increased flow levels and that more frequent flow experiences are associated with increased creativity [7]. These findings suggest that flow state may significantly impact both work performance and creativity. With the increasingly diverse work arrangements, such as remote and teleworking, desk-based tasks have increased, alongside heightened productivity and creativity demands. In this context, real-time detection of flow states in workers, combined with feedback, could enable workers to autonomously manage their productivity, task completion, and work hours. In addition, by visualizing their psychophysiological states, this approach could not only enhance productivity and motivation, but also contribute to better self-management of health and well-being.

Methods for assessing flow state typically involve questionnaires such as the Flow State Scale [8] and the Flow Experience Checklist [9]. In addition, specialized questionnaires have been developed for specific contexts, such as the Work-Related Flow Inventory [10] for occupational tasks and the Flow State Scale for Occupational Tasks [11] for low-demand activities in daily life. However, no clear criteria have been established to define flow state or quantitatively measure its levels in real time. To evaluate the flow state continuously, it is preferable to utilize autonomic nervous system activity indices, such as heart rate or respiration, which can be measured relatively easily using a wearable device and/or remote sensing method and allow for real-time assessment.

Several studies on flow and autonomic nervous system activity indices have examined their associations with heart rate metrics. For example, Manzano et al. [12] reported that during piano performances, flow occurred under conditions of high task difficulty, which was associated with a reduction in RR intervals and an increase in the low-frequency/high-frequency (LF/HF) ratio, suggesting the facilitation of sympathetic nervous system (SNS) activity. Similarly, Tian et al. [13] identified a relationship between flow and SNS activity in experimental environments involving PCs, indicating that heart rate data may serve as potential markers for states preceding the onset of flow. However, other studies have demonstrated an inverse U-shaped relationship between task difficulty and flow, with a corresponding inverse U-shaped pattern in the LF component of heart rate variability [14–16]. These findings indicate a lack of consistency in the literature regarding the relationship between flow and heart rate metrics.

Lacey’s model [17] proposes that cardiovascular response patterns differ depending on the type of stimulus or task. These patterns are categorized according to the brain’s processing of external stimuli or information [18]. For example, mental tasks requiring cognitive effort and sensory rejection, such as mental arithmetic or document preparation, are associated with a cardiac-dominant response pattern—referred to as the Pattern 1 response—characterized by an elevated heart rate. Conversely, tasks that require sustained attention to external stimuli, such as mirror tracing or embedded figures tests, elicit a vascular-dominant response pattern—the Pattern 2 response—as evidenced by peripheral vasoconstriction and reduced heart rate [19–21]. While existing research on flow and cardiovascular responses predominantly emphasizes increased heart rate as a characteristic of flow, mirror-drawing tasks, in contrast, elicit heart rate deceleration. However, task conditions involving time pressure and competitive settings can induce a deep flow state with pronounced bradycardia and vasoconstriction [22]. This implies Pattern 2 response-inducing tasks may enhance the intensity of flow states. Consequently, flow state and Pattern 2 responses may have a strong association, with heart rate being a valuable index for assessing the flow state.

However, previous research has not investigated mental tasks characterized by sensory rejection under similar experimental conditions, nor considered their effects on Pattern 1 responses. As previously mentioned, during sensory rejection tasks, increased β-adrenergic sympathetic activity results in a cardiac-dominant response, characterized by an elevated heart rate due to increased cardiac output [21]. If flow state level can be inferred from the heart rate responses, a significant heart rate acceleration, identified as a Pattern 1 response, may occur during flow state. Furthermore, independent of the previously mentioned findings, prior research has documented the emergence of a frontal midline theta rhythm—Fm theta—during the Kraepelin test [23]. Fm-theta activity occurs when individuals maintain sustained cognitive engagement with a task [24], suggesting that the Uchida-Kraepelin (U-K) test [25] may induce flow state. Therefore, this study employed the U-K test, a mental arithmetic task, to investigate the relationship between flow state and autonomic nervous system indices, including heart rate metrics.

## Materials and methods

### Participants

This study involved 18 healthy male participants aged over 18 years (mean age: 21.9 ± 0.3 years). All participants were nonsmokers, free from medication, and without any cardiovascular diseases such as arrhythmia or hypertension. This study was approved by the Ethics Committee of Medical Research, University of Occupational and Environmental Health, Japan (Approval No. R3-059). Participant recruitment for this study began at the University of Occupational and Environmental Health from November 29, 2021, and the experiment was completed by January 31, 2022. All participants provided written informed consent after receiving a detailed explanation of the study. A report on the completion of the study was submitted on March 31, 2024.

### Experimental procedure

**Fig 1:**
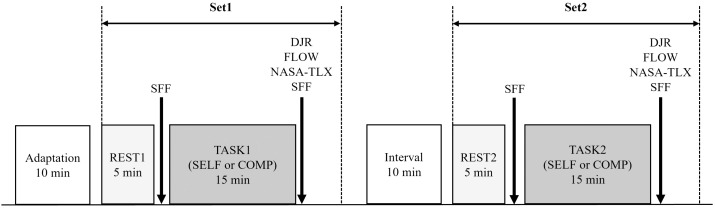
Experimental procedure. *Notes:* SFF = Subjective feelings of fatigue; DJR = Duration judgment ratio; FLOW = 12-item scale adaped from the Flow State Scale [11] and its Japanse version [9]; NASA-TLX = National Aeronautics and Space Administration Task Load Index; REST1 and REST 2 = 5-min rest periods in the first and second sets, respectively; TASK1 and TASK2 = tasks in the first and second sets, respectively. The order of SELF and COMP conditions was randomized among participants.

The experiment was conducted in a soundproof room with a floor area of 7.63 m^2^ (290 × 263 cm) and a height of 271 cm. Prior to the experiment, the participants received an explanation of the experimental procedures and practiced the task. Subsequently, electrodes and sensors were attached, and the participants were allowed to acclimate to the experimental environment by sitting quietly for approximately 10 min. The experimental procedure (Fig 1) involved two sets, with a 5-min rest period (REST1 or REST2), followed by the U-K test for 15 min in each set. The task conditions varied and a 10-min rest interval was provided between sets. After the rest period and task completion, subjective feelings of fatigue (SFF) were assessed using the Jikaku-sho Shirabe [26]. In addition, the Flow Experience Checklist (FLOW) [9,11], duration judgment ratio (DJR) [27], and National Aeronautics and Space Administration Task Load Index (NASA-TLX) [28] were administered after each task.

### Experimental task

The U-K Test was used as the sensory rejection task. Although this test is primarily used to assess individual personality traits, it is widely utilized for evaluating psychological states such as stress and concentration [25]. The participants were instructed to perform a single-digit addition of adjacent numbers on a specialized test sheet, switching to a new row every minute on the experimenter’s cue, for 15 min per set. The task conditions were as follows: 1) the task to be performed at own pace (SELF) and 2) to be performed according to the following instruction, “Always do as many calculations as possible, aiming to exceed the preceding performance,” thereby encouraging self-competition (COMP). The target performance in the first row of the COMP condition was set to 60 per min based on our previous study, where participants were instructed to increase the number of calculations in each successive row (i.e., perform more in the second row than in the first row). The order of the task conditions was randomized across participants. For task performance, the total number of additions and correct rate over the 15-min period were calculated for each condition.

### Subjective assessment

FLOW (12 questions, simplified version) was administered with reference to the Flow State Scale [11] and its Japanese version [9]. Participants responded to the 12 items using a 7-point Likert scale (1 = strongly disagree, 2 = disagree, 3 = slightly disagree, 4 = undecided, 5 = slightly agree, 6 = agree, and 7 = strongly agree). The following four factors: “clear goals,” “unambiguous feedback,” “sense of time,” and “balance between challenges and skills” were obtained from the average scores of the three items. DJR [27] was used to assess time perception by asking whether the actual task duration (15 min) felt shorter or longer on a visual analog scale (from 0: shortest to 100: longest). NASA-TLX [28], comprising six subscales of mental demand (MD), physical demand (PD), temporal demand (TD), own performance (OP), effort (EF), and frustration (FR), was used to assess subjective mental workload. The participants rated each subscale using a visual analog scale (VAS), with scores ranging from 0 to 100. The weighted mean of the six NASA-TLX subscales—the adaptive weighted workload (AWWL) [29]—was calculated without paired comparisons. SFF were assessed using the Jikaku-sho Shirabe [26], Participants rated the intensity of each symptom using a 5-point scale: “not at all,” “slightly,” “moderately,” “considerably,” and “extremely,” corresponding to scores of 1–5, respectively. These 25 items were categorized into five factors: (i) drowsiness, (ii) instability, (iii) uneasiness, (iv) local pain or dullness (dullness), and (v) eyestrain.

### Physiological measurement

Electrocardiogram (ECG), photoelectric plethysmography (PTG), tissue blood flow (TBF), tissue blood volume (TBV), and skin potential level (SPL) were obtained. ECG signals were recorded from the chest-manubrium 5 (CM_5_) lead at a 1 kHz sampling rate (SYNAFIT 2200, NEC San-ei Instruments Ltd., Tokyo, Japan), and RR intervals and heart rate (HR) were derived. The power spectra of the RR intervals were calculated using autoregressive power spectral analysis. The LF and HF components of the heart rate variability (HRV) spectra were extracted and the LF/HF ratio was calculated. PTG was measured from the second digit of the left hand using a calibrated plethysmograph (Nihon Kohden MLV-2301), and the PTG amplitude (PTG amp) was calculated from the difference between the peak and trough of each pulse. The TBF and TBV were assessed using a laser Doppler blood flow meter (OMEGAWAVE, OMEGA FLOW, FLO-C1) with sensors attached to the nasal tip. Changes in TBV and TBF primarily reflect vasoconstriction and vasodilation, respectively, and are indices for evaluating autonomic nervous system activity. For example, in the relationship between forehead skin blood flow and autonomic nervous system activity, blood flow increases when parasympathetic nervous system (PNS) activity predominates [30]. SPL was recorded from the thenar eminence of the left palm using a DC amplifier (Nihon Kohden AD-641G) with the forearm as the reference site. Regarding the psychophysiological interpretation of the SPL, when the reference electrode is placed on the forearm as the positive electrode, an increase in the arousal level results in a deep negative shift in the SPL waveform, whereas a decrease causes the value to approach zero [31].

### Statistical analysis

Two of the 18 participants were excluded from the analysis because of frequent artifacts during measurements and another was excluded, due to experiencing frequent arrhythmias, resulting in a final sample size of 15. For subjective assessments, mean values and standard errors were calculated across flow scores, time perception scores (DJR), NASA-TLX subscales, AWWL, and the five categories of the SFF. One-way repeated measures analysis of variance (ANOVA) was conducted to compare SFF scores across the four blocks (REST1, SELF, REST2, and COMP). Greenhouse-Geisser corrections were applied to the degrees of freedom, and multiple comparisons were performed using the Tukey-Kramer method when the main effect was significant. For other subjective measures, paired t-tests were used to compare the differences between task conditions in terms of subjective measures and task performance.

The 15-min task period was divided into three 5-min blocks (TK1, TK2, and TK3) to assess changes in physiological responses during the tasks under each condition. Standardized scores were calculated for eight blocks for each participant (condition [2 levels]: SELF and COMP × block [4 levels]: REST, TK1, TK2, and TK3). Statistical analysis was conducted using a two-way repeated-measures ANOVA with task condition (two levels) and block (four levels) as the factors. Repeated-measures one-way ANOVA across all eight blocks, followed by Tukey-Kramer post-hoc tests, were performed for measures with significant interactions (SPSS Statistics ver. 25, IBM Corp., Armonk, NY, USA). The significance level was set at p < 0.05.

## Results

**Fig 2:**
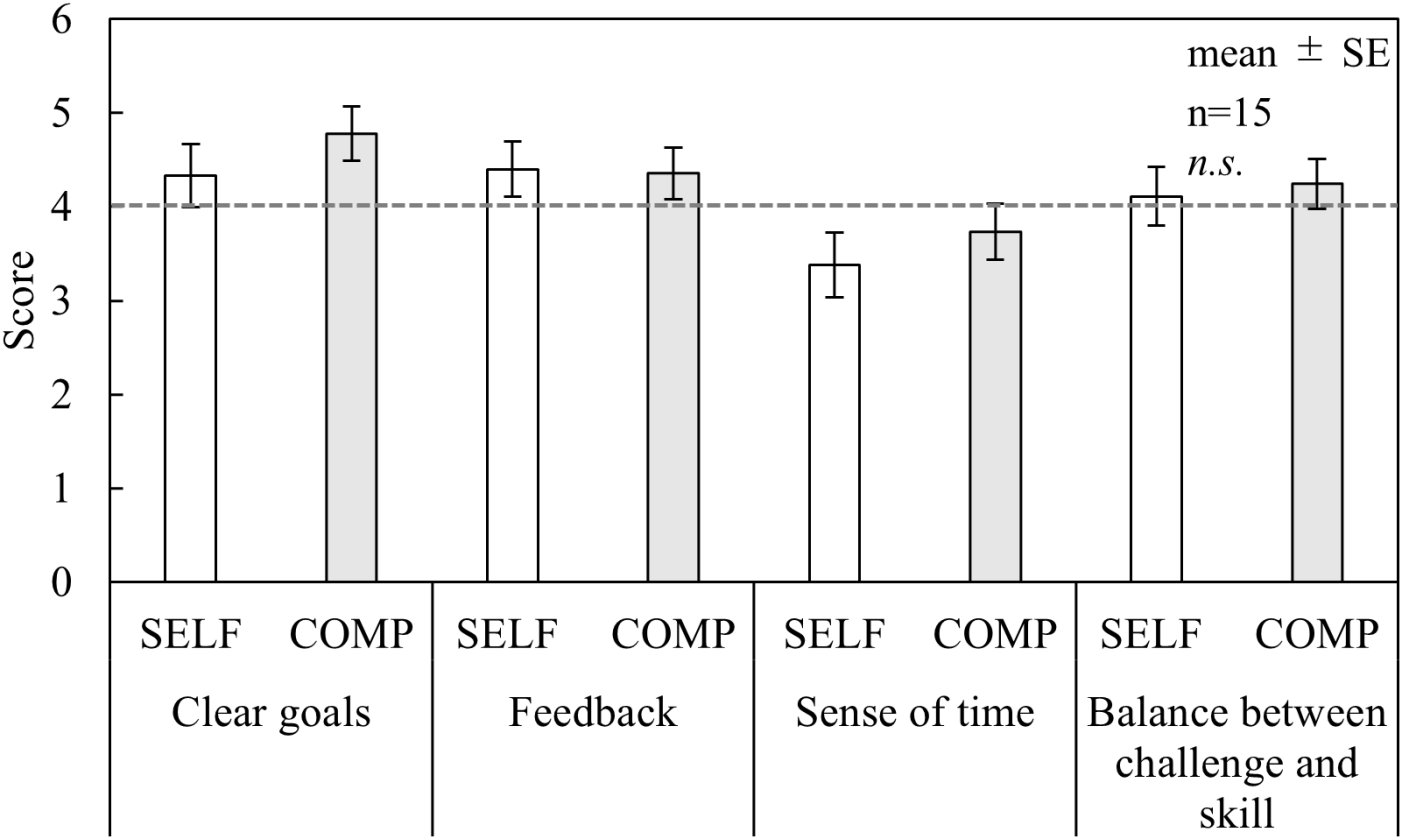
Flow scores. *Notes:* SELF = self-paced condition; COMP = competitive condition; SE = standard error; n.s. = non-significant. A dashed line at 4.0 on the vertical axis indicates the threshold used to identify flow experience. This figure is based on the version originally included in the first author’s doctoral dissertation (Kyushu Institute of Technology, 2023), available at https://kyutech.repo.nii.ac.jp/records/2000123. Statistical values have been updated following reanalysis using a unified sample size (n = 15), and minor formatting adjustments were applied for consistency with journal style.

**Fig 3:**
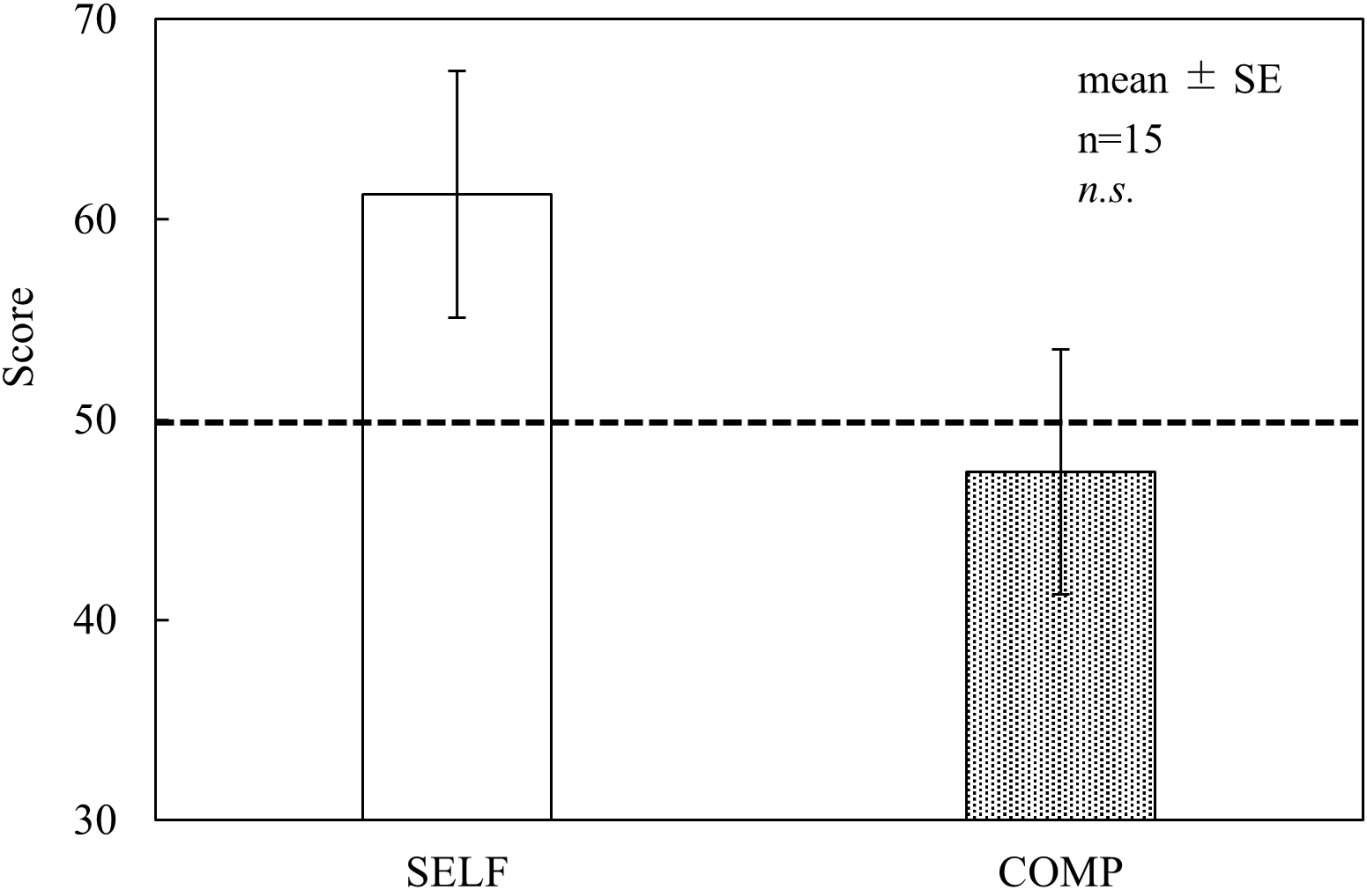
Duration judgment ratio (DJR) results. *Notes:* A score of 50, indicated by the dashed line, implies that the participants’ subjective perception of time matched the actual task duration. SELF = self-paced condition; COMP = competitive condition; SE = standard error; n.s. = non-significant. This figure is based on the version originally included in the first author’s doctoral dissertation (Kyushu Institute of Technology, 2023), available at https://kyutech.repo.nii.ac.jp/records/2000123. Statistical values have been updated following reanalysis using a unified sample size (n = 15), and minor formatting adjustments were applied for consistency with journal style.

**Fig 4:**
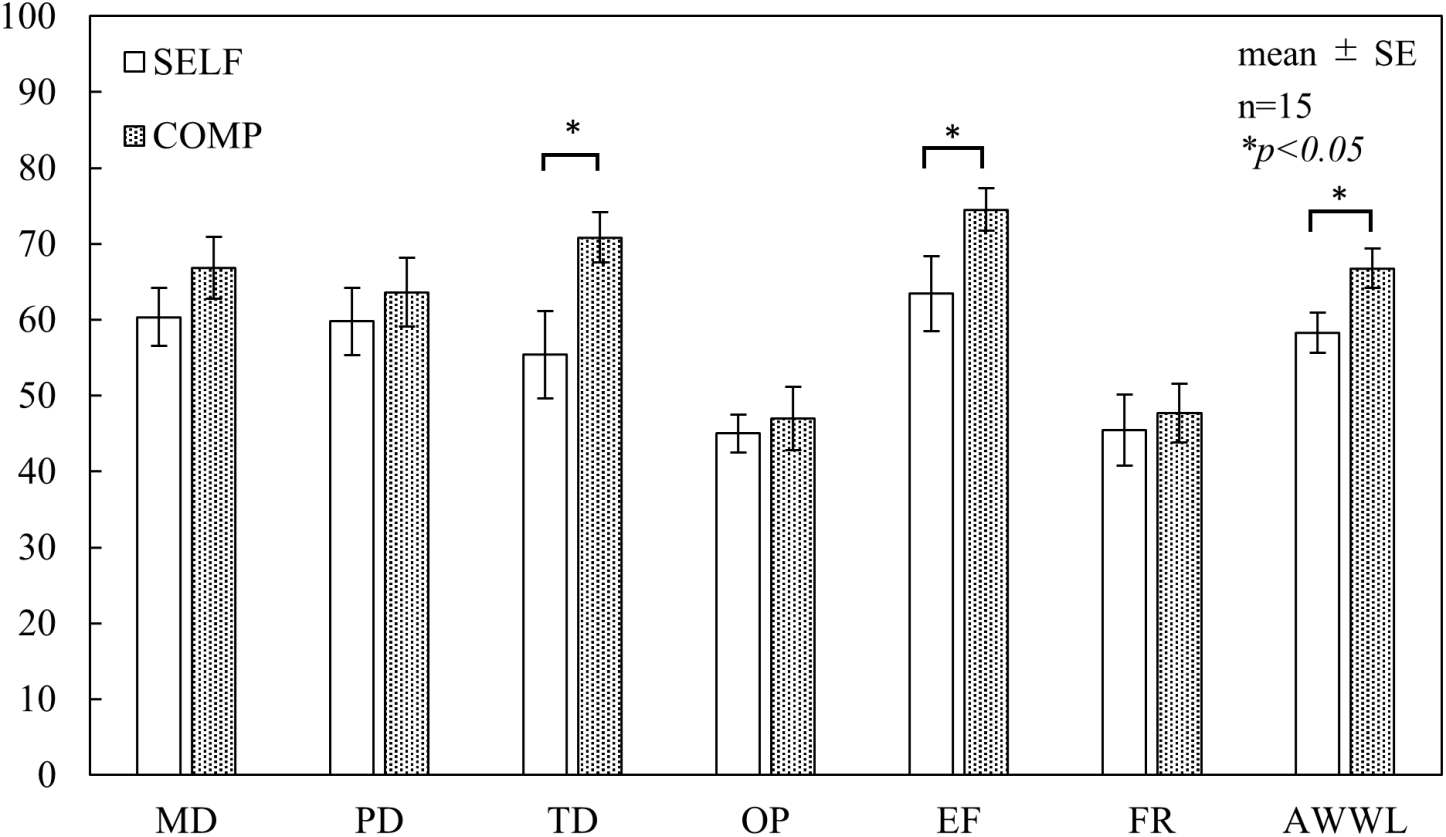
NASA-TLX scores. *Notes:* NASA-TLX = National Aeronautics and Space Administration Task Load Index; SELF = self-paced condition; COMP = competitive condition; SE = standard error; MD = mental demand; PD = physical demand; TD = temporal demand; OP = own performance; EF = effort; FR = frustration; AWWL = adaptive weighted workload. This figure is based on the version originally included in the first author’s doctoral dissertation (Kyushu Institute of Technology, 2023), available at https://kyutech.repo.nii.ac.jp/records/2000123. Statistical values have been updated following reanalysis using a unified sample size (n = 15), and minor formatting adjustments were applied for consistency with journal style.

**Fig 5:**
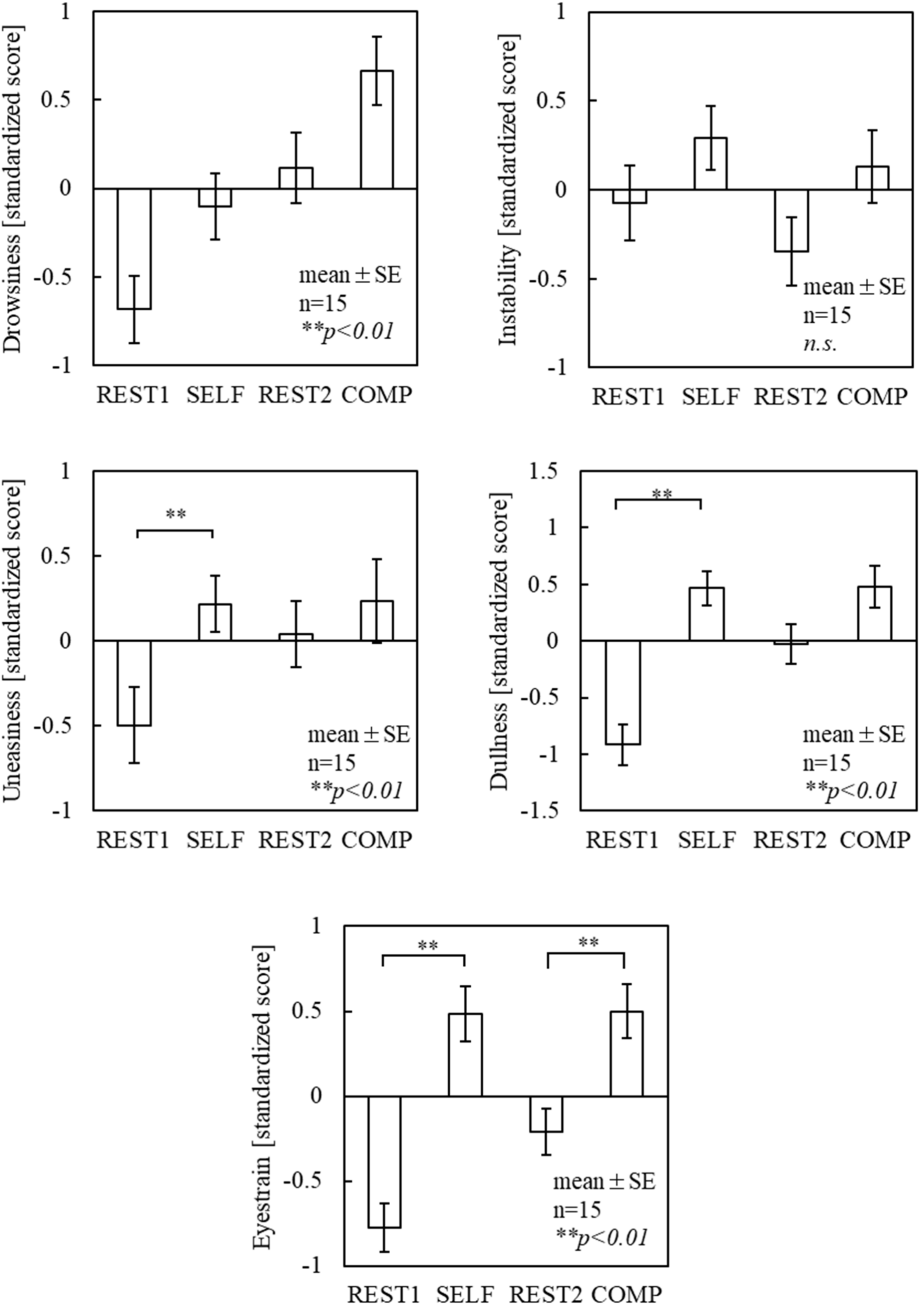
SFF scores. *Notes:* SFF = subjective feelings of fatigue; REST1 and REST2 = 5-min rest periods in the first and second sets, respectively; SELF = self-paced condition; COMP = competitive condition; SE = standard error. This figure is based on the version originally included in the first author’s doctoral dissertation (Kyushu Institute of Technology, 2023), available at https://kyutech.repo.nii.ac.jp/records/2000123. Statistical values have been updated following reanalysis using a unified sample size (n = 15), and minor formatting adjustments were applied for consistency with journal style.

**Fig 6:**
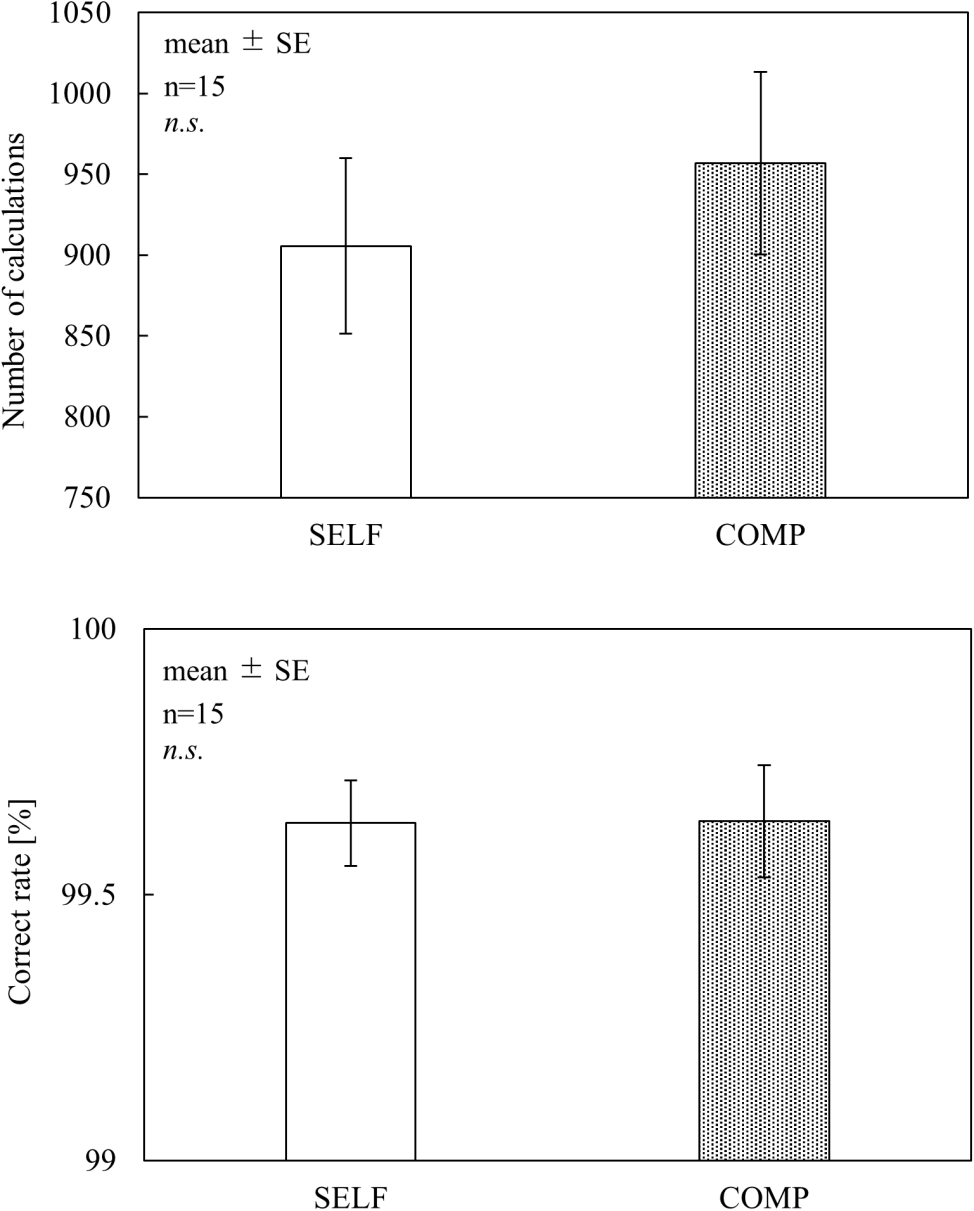
Task performance. *Notes:* SELF = self-paced condition; COMP = competitive condition; n.s. = non-significant; SE = standard error. This figure is based on the version originally included in the first author’s doctoral dissertation (Kyushu Institute of Technology, 2023), available at https://kyutech.repo.nii.ac.jp/records/2000123. Statistical values have been updated following reanalysis using a unified sample size (n = 15), and minor formatting adjustments were applied for consistency with journal style.

As shown in Fig 2, no significant differences in flow scores were found between the conditions across all subscales. Regarding the DJR results (Fig 3), the COMP condition exhibited lower scores than the baseline (50 points) for time perception; however, no significant differences were observed between the conditions. In the NASA-TLX results (Fig 4), TD and EF scores were significantly higher in the COMP condition than in the SELF condition (TD, p < 0.05; EF, p < 0.05). No significant differences were found between the conditions for the other subscales. AWWL was significantly higher in the COMP condition than in the SELF condition (p < 0.05). The SFF results (Fig 5) indicated significant main effects of the blocks in drowsiness, uneasiness, dullness, and eyestrain (p < 0.01). The uneasiness and dullness scores were significantly higher in the SELF condition (uneasiness, p < 0.01; dullness, p < 0.01), and the eyestrain scores were significantly higher in both conditions (SELF, p < 0.01; COMP, p < 0.01), than in the preceding rest periods (REST1 and REST2). Regarding task performance (Fig 6), although more participants in the COMP condition showed an increase in task output than those in the SELF condition, no significant differences were found between the conditions. The correct rates were close to 100% in both conditions, with no significant differences.

**Fig 7:**
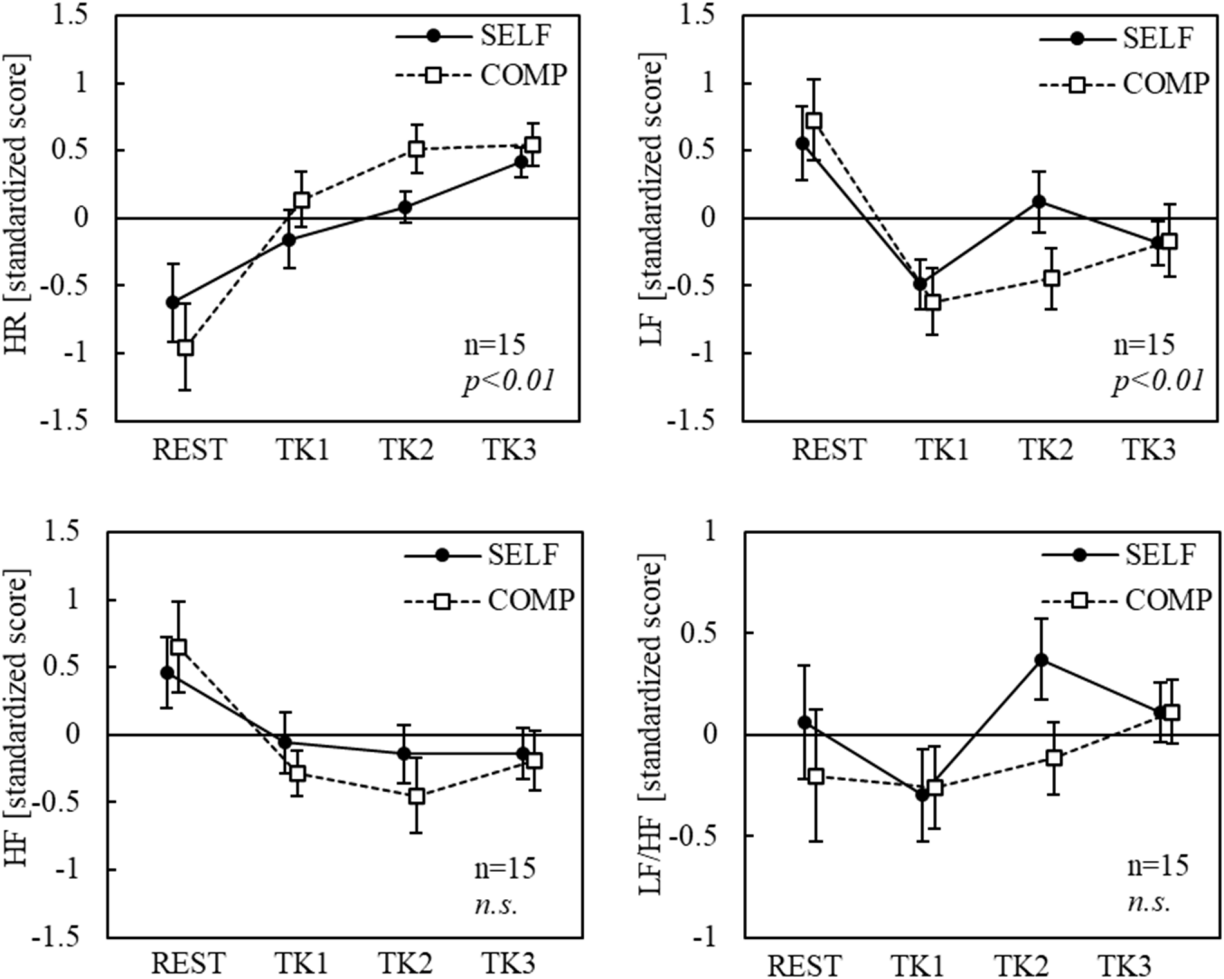
Changes in HR and HRV indices. *Notes:* Bars indicate standard errors of the mean. HR = heart rate; HRV = heart rate variability; HF = high frequency; LF = low frequency; SELF = self-paced condition; COMP = competitive condition; REST = rest period; TK1, TK2, TK3 = three 5-min blocks, respectively; n.s. = non-significant. Reproduced with minimal modification from the first author’s doctoral dissertation (Kyushu Institute of Technology, 2023), available at https://kyutech.repo.nii.ac.jp/records/2000123.

The HR and HRV indices are shown in Fig 7. While block had a significant main effect (p < 0.01) on the HR, condition did not. In the SELF condition, the HR was significantly higher during TK3 than during REST. In the COMP condition, the HR was significantly higher during all task blocks than during REST. While block had a significant main effect (p < 0.01) on the LF, condition did not. In the SELF condition, the LF was significantly lower during TK1 and TK3 than during REST. In the COMP condition, the LF was significantly lower during TK1 and TK2 than during REST. Regarding HF, block had a significant main effect (*p <* 0.01), but condition did not. While no significant differences were found in the SELF condition, the HF in the COMP condition was significantly lower during TK2 than during REST. No significant main effects of condition or block were found for LF/HF.

**Fig 8:**
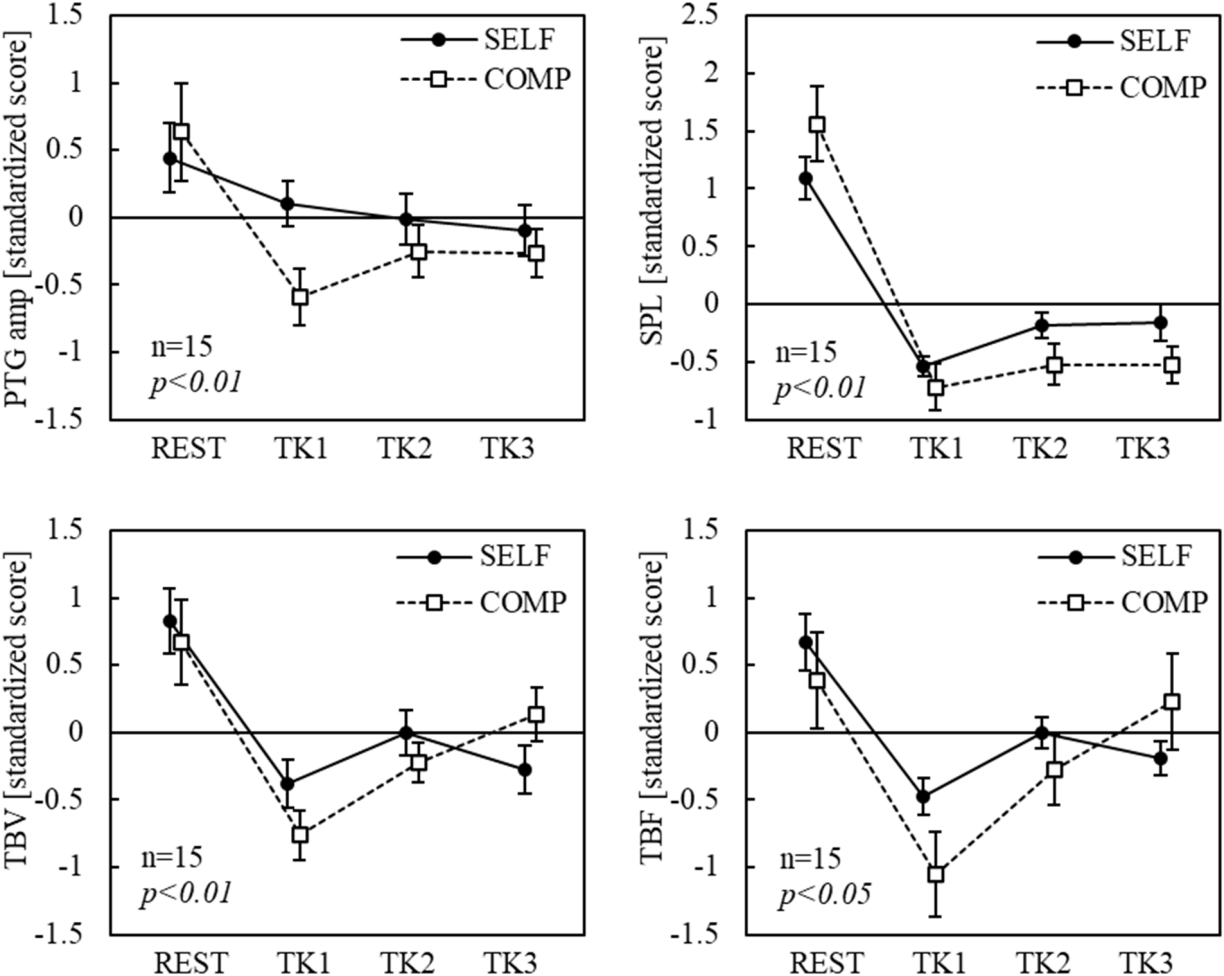
Changes in other ANS indices. *Notes:* Bars indicate standard errors of the mean. ANS = autonomic nervous system; SELF = self-paced condition; COMP = competitive condition; TBV = tissue blood volume; TBF = tissue blood flow; SPL = skin potential level; PTG amp = photoelectric plethysmography amplitude; REST = rest period; TK1, TK2, TK3 = three 5-min blocks, respectively. Reproduced with minimal modification from the first author’s doctoral dissertation (Kyushu Institute of Technology, 2023), available at https://kyutech.repo.nii.ac.jp/records/2000123.

The results for PTG amp, SPL, TBV, and TBF are shown in Fig 8. The PTG amp showed no significant main effect of condition, but a significant main effect of block (p < 0.01). Compared with the pre-task rest period, pulse wave amplitude reduced significantly during TK1 in the COMP condition. No interactions were observed in the PTG amp. SPL showed a significant main effect of block (p < 0.01), but not of condition. In both conditions, SPL was significantly lower in all task blocks, indicating more perspiration, than in REST. No interactions were observed. While block had a significant main effect on TBV (p < 0.01), condition did not. In the SELF condition, TBV was significantly lower, indicating peripheral vasoconstriction during TK1 than during REST, whereas in the COMP condition, TBV was significantly lower during TK1 and TK2 than during REST. No interactions were observed. The TBF showed no significant main effect of condition, but that of block was observed (p < 0.05). In the SELF condition, TBF was significantly lower during all TK blocks than during REST, and in the COMP condition, a significant reduction was observed during TK1. No interactions were observed.

## Discussion

In this study, the participants performed mental arithmetic tasks classified as sensory rejection tasks. Based on the findings of our previous research [22], task instructions were designed to maintain participants’ motivation by setting clear goals and allowing them to monitor their progress. However, in the SELF condition, 12 of the 15 participants had mean scores below 4.0 points (“neither agree nor disagree”) on the “sense of time transformation” subscale of the Flow State Scale (FSS). Previous studies have suggested that scores at or above the midpoint of the rating scale may be used as a criterion for identifying flow experience [32,33]. As the FSS in this study used a 7-point Likert scale, we adopted 4.0 points as a minimum threshold for defining flow. This suggests that the arithmetic task using the U-K test might not have effectively induced a flow state. In addition, the absence of significant differences in flow scores across all subscales between conditions indicates that the COMP condition might have failed to elicit a flow state.

The lack of significant differences in task performance across the task conditions suggests that task control through instructional manipulation is challenging. The U-K test, a simple single-digit addition task, has inherently low difficulty and is monotonous. This is supported by the average score of 4.4 points (below neutral point) for the skill-related question, “I felt that I had the skills required at that time” in the flow evaluation, indicating that participants did not perceive it as difficult. The results of the NASA-TLX suggested that task engagement was well controlled, as indicated by the TD scores. Additionally, the EF scores were higher in the COMP condition (SELF condition: 63.4; COMP condition: 74.5), indicating a significant difference in AWWL, with a higher overall workload in the COMP condition. While eyestrain increased significantly in both conditions, the elevated workload under the COMP condition may reflect greater mental effort and time pressure resulting from the competitive nature of the task. These factors may have interfered with the flow experience or limited improvements in task performance. Consequently, it is suggested that low-difficulty arithmetic tasks such as the U-K test may not induce a flow state even when the task difficulty is increased through competition. To effectively induce a flow state during the U-K test, it is important to integrate the elements of active coping that facilitate sustained attentional control, enabling participants to focus on error avoidance. This requires task conditions that provide continuous performance feedback, not only on task output but also on accuracy, to ensure that cognitive and psychophysiological engagements are maintained throughout the task.

Although the subjective state results suggest that a flow state may not have been induced in this experiment, human emotions and psychological states may fluctuate continuously during task execution with corresponding changes in physiological responses. Therefore, the 15-min task period was divided into three 5-min intervals in each condition, and the physiological indices during these intervals were analyzed. The results showed a significant increase in HR from rest to the task period under both conditions, suggesting a Pattern 1 response. However, the blocks with significant differences varied between conditions. Under the SELF condition, a significant increase in HR from the pre-task rest period was observed during TK3, which occurred 10–15 min after the task began. The gradual increase in HR from TK1 to TK3 suggests the potential effect of task-related fatigue. However, previous studies have shown that HR does not change during repeated mental arithmetic tasks performed for 10 min across six sets [34]. Therefore, the increase in HR may have been caused by transient stress or tension rather than fatigue. In contrast, the COMP condition showed a remarkable increase in HR during the first 5 min of the task (TK1). Regarding vascular indices, both TBV and TBF significantly decreased between the resting period and TK1 in both conditions, and PTG amp significantly decreased in the COMP condition. Additionally, the significant decrease in SPL, implying more perspiration, in TK1 than in the preceding resting period suggests that the task load activated α-sympathetic nervous system activity, leading to peripheral vasoconstriction. These findings are consistent with those of previous studies that examined the effects of mental arithmetic tasks but not specifically the Kraepelin test [34]. The HR changes of the participants who exhibited the largest changes in flow and time perception scores were analyzed. Although the number of participants was small (n = 3), all showed a decrease in HR during TK1. Our previous research [22] suggested that these three participants exhibited Pattern 2 responses. However, further investigation is necessary because these individuals might have exhibited a predisposition toward vascular-dominant autonomic responses. Furthermore, these response patterns are evident in how individuals cope with stimuli [21]. For example, Pattern 1 responses occur in situations in which sustained attention is required for active coping, involving deliberate efforts to address the stimulus. In contrast, pattern 2 responses were more likely to occur in unavoidable stimulus conditions, necessitating continuous monitoring, as seen in passive coping. Previous research has demonstrated that flow state is associated with active coping [12], suggesting that the three participants who exhibited a Pattern 2 response are unlikely to have engaged in passive coping. Thus, several factors must be considered in the relationship between flow state and response patterns, necessitating further investigations.

Regarding HRV indices, a decrease in the LF was observed from the pre-task rest period to the task duration under both conditions, suggesting the inhibition of PNS activity. This decrease was particularly pronounced under the COMP conditions. Recent studies have questioned the reliability of HRV indices derived from spectral analysis as indicators of autonomic nervous system activity related to psychological states, particularly highlighting the challenges in interpreting the LF/HF ratio [35,36]. These reports suggest that the LF component is more closely associated with PNS activity than with SNS activity, leading to inconsistent changes in the LF/HF ratio. In this study, no significant changes were observed in the HF components of HRV, which are commonly used as PNS activity indices. HF is sensitive to respiratory frequency, and it is necessary to control the respiratory rate during assessments to ensure accuracy [12]. Additionally, reports have highlighted the inconsistencies in the association between HF and PNS activity [37]. Given the variability and challenges in interpreting HRV indices, the estimation of flow states based solely on the autonomic nervous system activity derived from HR may be problematic. Moreover, although previous studies have examined correlations among autonomic and vascular indices, such relationships are often inconsistent due to the local specificity and directional fractionation of autonomic responses [17,38]. Therefore, correlation analyses were not included in the present study, as they could complicate interpretation rather than clarify it. Instead, we focused on condition- and time-dependent trends in each physiological measure.

This study provided valuable insights into the relationship between flow state and autonomic response patterns using a mental task in a controlled laboratory setting with young male participants aged 18 years and older. However, to apply these findings to real-world settings, further validation in environments such as workplaces and schools is required, using tasks that closely resemble practical work activities. While certain tasks, such as manual vehicle control and remote manipulation in robotic surgery (e.g., Da Vinci system), are explicitly characterized by sensory intake processes, the majority of real-world tasks are likely to involve a combination of both sensory intake and sensory rejection components.

Addressing this issue would require long-term recording of individual behavioral information and physiological data, potentially utilizing AI techniques. Indeed, the evaluation of physical activity using big data is advancing, and it is possible to consider the influence of individual characteristics such as gender, age, and personality traits. However, even in real-world work settings, it is difficult to identify physiological indices that reflect various psychological states without controlling task conditions such as task type and duration. Therefore, a key challenge in long-term studies exploring physiological indices to detect flow state will be determining how to constrain individual data. Additionally, a fundamental issue in determining the flow state lies in the absence of clear criteria for scores derived from subjective evaluations. To resolve these challenges, further foundational research is required, involving long-term and multifaceted investigations that incorporate subjective assessments such as time perception evaluations used in this study, task performance, and physiological indicators, including central nervous system activity.

## Conclusion

In this study, participants performed a mental arithmetic task—a sensory rejection task—to induce a flow state. However, flow state was not induced even when instructions met the prerequisites for flow in the low-difficulty U-K test. Previous studies have indicated that real-time feedback on accuracy and workload during mental arithmetic tasks does not improve concentration. Therefore, tasks such as mirror tracing [22], which are not only more difficult but also incorporate elements of gamification and allow real-time visualization of skill improvement, have a higher likelihood of inducing a flow state. It is likely that many complex tasks possess the characteristics of both sensory rejection, as observed in the U-K test, and sensory intake associated with the mirror-tracing task. However, owing to the wide variability in task types, it is impractical to conduct experimental investigations across all possible tasks. Therefore, as demonstrated in this study, setting up experimental conditions that reflect the “extreme ends” of sensory rejection tasks is a crucial initial step in understanding the psychophysiological mechanisms underlying flow and its potential application in real-world settings.

Some participants with increased flow scores exhibited a decreased HR, supporting the association between Pattern 2 responses and flow state. Although HR changes alone may estimate the flow state level, detecting the flow state based on a single index is challenging. Considering the established relationship between flow and Pattern 2 responses, it is recommended to assess flow using multiple physiological markers, including pulse wave velocity and blood pressure. Additionally, as an index of the central nervous system, the Fm theta may be associated with flow state [39,40]. The combined use of Fm-theta with autonomic nervous system measures may enhance the sensitivity and accuracy of flow state detection.
